# Time-Course of Alterations in the Endocannabinoid System after Viral-Mediated Overexpression of α-Synuclein in the Rat Brain

**DOI:** 10.3390/molecules27020507

**Published:** 2022-01-14

**Authors:** Rachel Kelly, Alexis-Pierre Bemelmans, Charlène Joséphine, Emmanuel Brouillet, Declan P. McKernan, Eilís Dowd

**Affiliations:** 1 Department of Pharmacology & Therapeutics and Galway Neuroscience Centre, National University of Ireland Galway, H91 W5P7 Galway, Ireland; r.kelly35@nuigalway.ie (R.K.); declan.mckernan@nuigalway.ie (D.P.M.); 2 Neurodegenerative Diseases Laboratory, Molecular Imaging Research Center, Paris-Saclay University, CEA, CNRS, F-92265 Fontenay-aux-Roses, France; alexis.bemelmans@cea.fr (A.-P.B.); charlene.josephine@cea.fr (C.J.); emmanuel.brouillet@cnrs.fr (E.B.)

**Keywords:** Parkinson’s, neuroinflammation, cannabinoids, alpha-synuclein

## Abstract

Since the discovery of α-synuclein as the major component in Lewy bodies, research into this protein in the context of Parkinson’s disease pathology has been exponential. Cannabinoids are being investigated as potential therapies for Parkinson’s disease from numerous aspects, but still little is known about the links between the cannabinoid system and the pathogenic α-synuclein protein; understanding these links will be necessary if cannabinoid therapies are to reach the clinic in the future. Therefore, the aim of this study was to investigate the time-course of alterations in components of the endocannabinoid system after viral-mediated α-synuclein overexpression in the rat brain. Rats were given unilateral intranigral injections of AAV-GFP or AAV-α-synuclein and sacrificed 4, 8 and 12 weeks later for qRT-PCR and liquid chromatography–mass spectrometry analyses of the endocannabinoid system, in addition to histological visualization of α-synuclein expression along the nigrostriatal pathway. As anticipated, intranigral delivery of AAV-α-synuclein induced widespread overexpression of human α-synuclein in the nigrostriatal pathway, both at the mRNA level and the protein level. However, despite this profound α-synuclein overexpression, we detected no differences in CB_1_ or CB_2_ receptor expression in the nigrostriatal pathway; however, interestingly, there was a reduction in the expression of neuroinflammatory markers. Furthermore, there was a reduction in the levels of the endocannabinoid 2-AG and the related lipid immune mediator OEA at week 12 post-surgery, indicating that α-synuclein overexpression triggers dysregulation of the endocannabinoid system. Although this research does show that the endocannabinoid system is impacted by α-synuclein, further research is necessary to more comprehensively understand the link between the cannabinoid system and the α-synuclein aspect of Parkinson’s disease pathology in order for cannabinoid-based therapies to be feasible for the treatment of this disease in the coming years.

## 1. Introduction

Parkinson’s disease is a chronic progressive neurodegenerative disease that manifests primarily clinically as a motor disorder, with patients presenting with bradykinesia, resting tremors, rigidity and postural instability [1]. Pathologically, Parkinson’s disease is characterised by the degeneration of the nigrostriatal dopaminergic neurons and the formation of intracellular eosinophilic inclusions termed Lewy bodies, the main component of which is α-synuclein protein [2,3]. α-synuclein is a 140 amino acid protein that is abundantly expressed in the brain, especially in the substantia nigra, and is found extensively in presynaptic terminals [4]. While the normal physiological role of α-synuclein remains to be definitively elucidated, its location in nerve terminals and results from animal studies suggest that it has a role in synaptic vesicle trafficking and dopamine signalling [5].

Since the discovery of α-synuclein in Lewy bodies in 1997, it has become increasingly evident that this protein plays a crucial role in the neuropathology of Parkinson’s disease. Several mutations in the *SNCA* gene that encodes α-synuclein have been associated with familial inherited forms of Parkinson’s disease, as has the carrying of multiple copies of the gene such as duplications and triplications [6,7,8,9,10,11]. Furthermore, the conformation of α-synuclein appears to be important with regard to its toxicity. The protein undergoes extensive conformational transitions and misfolds to form oligomers, which aggregate into protofibrils and subsequently amyloid fibrils that form the basis of Lewy bodies [12]. Posttranslational modifications of the protein, for example, phosphorylation or ubiquitination, have also been implicated in α-synuclein aggregation and neurotoxicity [13]. Furthermore, an apparent correlation has been observed between the amount of insoluble α-synuclein, its location and the stage of the disease [14]. From these observations, the experimenters proposed six anatomical stages of Parkinson’s disease pathology, which concur with the development of disease symptoms. Moreover, α-synuclein was detected in the cells of foetal transplant grafts in Parkinson’s disease patients at post mortem, suggesting that α-synuclein is self-propagating, transmitting from cell to cell in a prion-like fashion [15]. It is clear that this protein is intricately linked to Parkinson’s disease progression and pathology.

The endocannabinoid system is a relatively newly discovered system in neuropharmacology. The two major receptors, CB_1_ and CB_2_, are both G-protein-coupled receptors coupled to inhibitory G proteins, with their downstream effects including inhibition of the enzyme adenylyl cyclase and activation of the mitogen-activated kinase pathway. The endocannabinoid ligands arachidonoyl ethanolamide (anandamide or AEA) and 2-arachidonoyl glycerol (2-AG) are synthesised “on demand” from their lipid precursors, and in neurons act as retrograde messengers upon their postsynaptic release. Exogenous cannabinoids, such as those found in *Cannabis* plants and synthetic cannabinoids, also act upon these cannabinoid receptors and modulate their activity [16]. Mounting evidence indicates that the endocannabinoid system has major functions in the pathophysiology of Parkinson’s disease. The CB_1_ receptor is highly expressed in the basal ganglia [17,18], indicating that this receptor may play a part in motor control. Indeed, locomotor suppression is a classical behavioural effect of CB_1_ receptor agonists in rodents [19]. CB_1_ knockout mice have more severe motor deterioration and neurodegeneration, as well as a reduced incidence of L-DOPA-induced dyskinesias compared to their wild-type counterparts [20]. In the acute 6-OHDA rat model of Parkinson’s disease, a decrease in CB_1_ receptor binding in the striatum was found in vivo using positron emission tomography and the CB_1_ receptor-specific radioligand [^18^F]MK-9470 [21]. Using the same neuroimaging approach in Parkinson’s disease patients, regional modifications of CB_1_ receptor availability were observed, which were strongly correlated with disturbances in cognitive function [22]. Furthermore, alterations in CB_1_ receptor expression have also been reported in post-mortem Parkinson’s disease patients [23,24,25,26], although this has been confounded by variability in therapeutic regimes and the disease course. Evidently, dysregulation of CB_1_ receptor levels and occupancy by endogenous ligands is linked to Parkinson’s disease pathophysiology.

On account of this functional role of the cannabinoid system in the basal ganglia circuitry, this system has been suggested as a potential target for the correction of the abnormal signalling occurring in the Parkinson’s disease brain. However, the complexity and intricacy of this system has led to some contrasting results, with anti-Parkinsonian effects exerted by drugs that both enhance and reduce endocannabinoid signalling [27,28,29,30,31]. Furthermore, the potential of cannabinoids to provide anti-Parkinsonian effects are being investigated via several alternative avenues, including as reducers of Parkinsonian drug-induced side effects, and as providers of disease-modifying effects [32].

Although α-synuclein is integral to the neuropathology of Parkinson’s disease, the associations between the endocannabinoid system and α-synuclein pathology have been poorly investigated to date. The first study to indicate a link between the cannabinoid system and α-synuclein examined CB_1_ receptor expression in the basal ganglia of α-synuclein knockout mice [33]. The researchers observed a biphasic response, with decreased CB_1_ expression and binding in the nigrostriatal pathway in young mice, but an elevation in older mice. Contrastingly, spontaneous deletion of the α-synuclein gene was associated with an increase in expression of the CB_1_ receptor in the hippocampus and amygdala in young mice in a later study [34]. Furthermore, overexpression of the A53T mutant form of α-synuclein that can cause a familial inherited form of Parkinson’s disease was associated with a reduction in CB_1_ receptor expression and binding in old mice [35]. In a pharmacological study in PC12 neuronal cells, the synthetic cannabinoid receptor agonist WIN 55,212-2 inhibited the cytoplasmic accumulation of α-synuclein and cell death induced by a proteasomal inhibitor [36]. In an astrocytic cell line, ACEA, a CB_1_ receptor agonist, prevented an α-synuclein-induced increase in proinflammatory cytokines [37].

Neuroinflammation is also a driver of the pathology of Parkinson’s disease, and both cannabinoids and α-synuclein have been shown to be linked to this neuropathological aspect. α-synuclein interferes with the phagocytic function [38,39] and inflammatory profile of microglia [39,40,41,42,43,44], although there is contrasting evidence regarding the different α-synuclein disease-related mutations and the disparate α-synuclein conformations. With regard to the cannabinoid system, agonists of the cannabinoid CB_2_ receptor have been shown to provide antineuroinflammatory effects and are thus being investigated as a potential therapeutic strategy [45]. This receptor is present on microglia in very low numbers in the healthy brain, but pronounced upregulation of this receptor has been observed in neurodegenerative disease patients [46,47,48], as well as in numerous animal models of such diseases [30,49,50]. However, thus far, it has not been investigated if alterations in the expression of this receptor or of other components of the endocannabinoid system are induced by α-synuclein overexpression.

Taking the aforementioned evidence into consideration, the aim of this study is to examine the time-course of changes in components of the endocannabinoid system after viral-mediated α-synuclein overexpression in the rat brain.

## 2. Results

### 2.1. Qualitative Visualisation of α-Synuclein Expression in the Nigrostriatal Pathway

**Figure 1:**
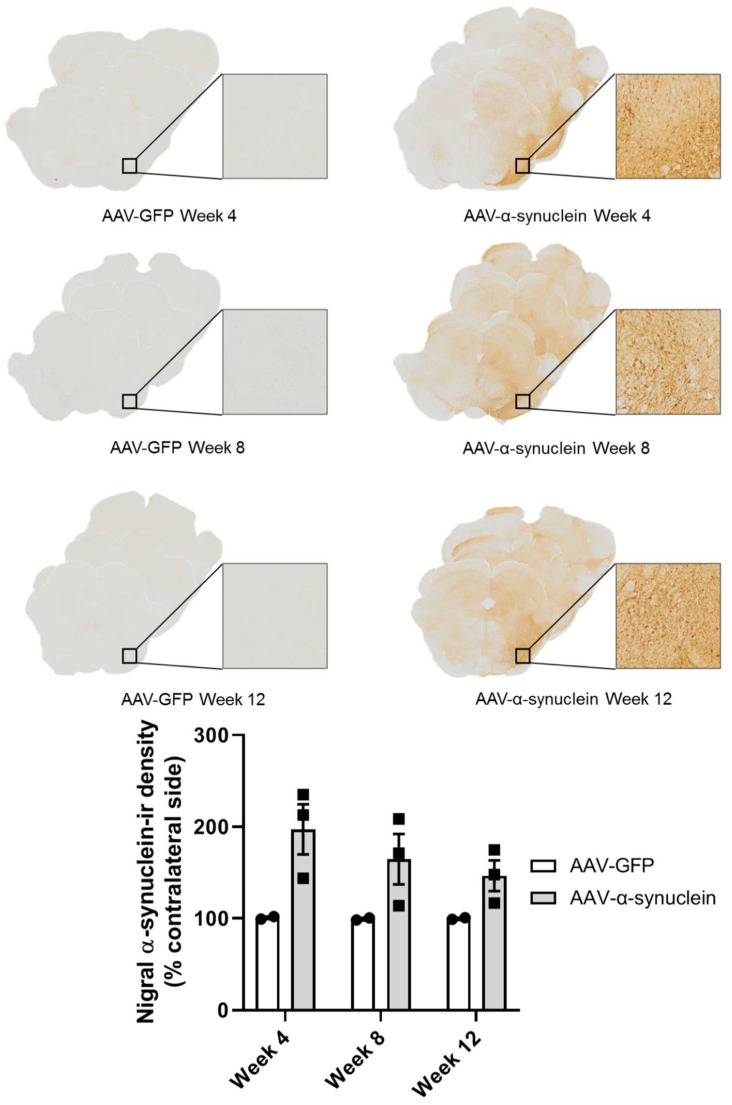
Expression of human α-synuclein in the substantia nigra after injection of AAV-α-synuclein. Top panel: photomicrographs showing α-synuclein expression in the substantia nigra at different time-points after the administration of the viral vectors. Bottom panel: graph showing the relative quantification of the expression of human α-synuclein in the substantia nigra using optical density image analyses of sections labelled by immunohistochemistry for human α-synuclein. Note that animals that received AAV-α-synuclein expressed human α-synuclein protein in the substantia nigra, but animals that received AAV-GFP did not. Data are represented as mean ± SEM with 2–3 animals per group.

**Figure 2:**
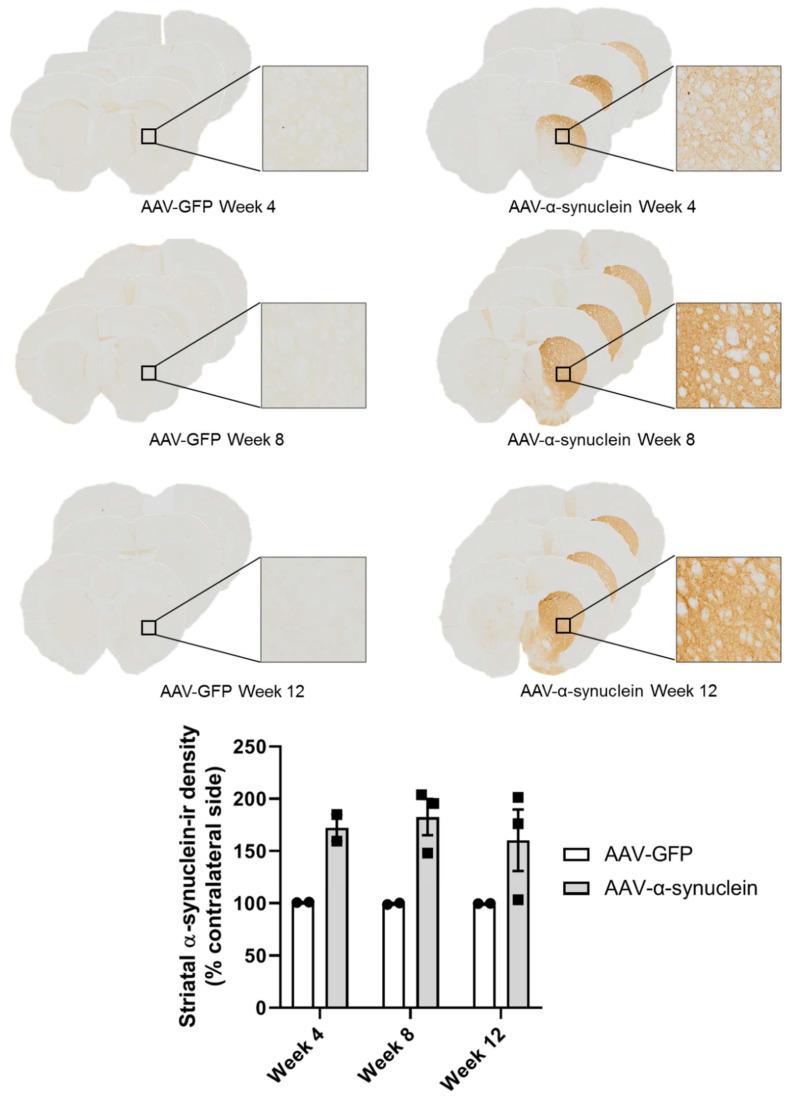
Expression of human α-synuclein in the striatum after injection of AAV-α-synuclein. Top panel: photomicrographs showing α-synuclein expression in the striatal terminals at different time-points after the intranigral administration of the viral vectors. Bottom panel: graph showing the relative quantification of the expression of human α-synuclein in the striatum using optical density image analyses of sections labelled by immunohistochemistry for human α-synuclein. Note that animals that received AAV-α-synuclein expressed human α-synuclein protein in the striatum, but animals that received AAV-GFP did not. Data are represented as mean ± SEM with 2–3 animals per group.

Post mortem, we firstly wished to visually confirm the presence of human α-synuclein in the nigrostriatal pathway. Immunohistochemical staining for human α-synuclein revealed that, as anticipated, intranigral administration of the adeno-associated viral vector overexpressing α-synuclein (AAV-α-synuclein), but not AAV-GFP (green fluorescent protein), induced visible staining of human α-synuclein protein in the substantia nigra (Figure 1) and across the midbrain. Similarly to the substantia nigra, α-synuclein staining was clearly evident in the nerve terminals of the striatum on the side of the brain ipsilateral to the lesion in the rats that received AAV-α-synuclein, but not in the animals that received the AAV-GFP control vector (Figure 2).

### 2.2. AAV-α-Synuclein Administration Led to Increased Expression of Human α-Synuclein

**Figure 3:**
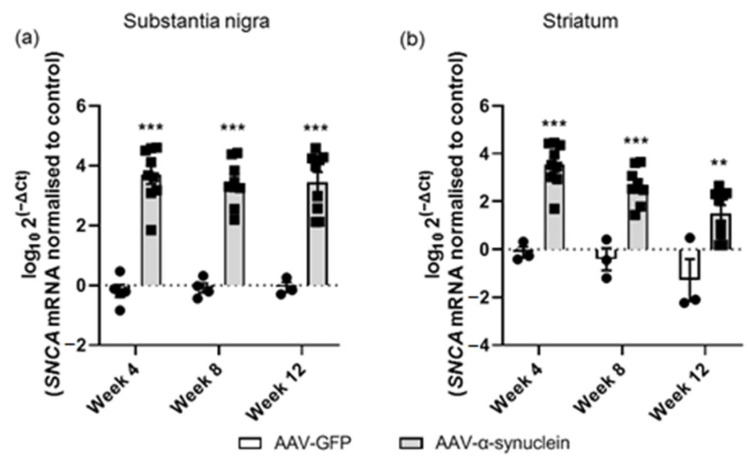
qRT-PCR analyses of human *α-synuclein* mRNA in the nigrostriatal pathway. The administration of AAV-α-synuclein induced a significant upregulation of human *α-synuclein* mRNA in (**a**) the substantia nigra and (**b**) in the striatum across all time-points. Data are represented as mean ± SEM with 3–9 animals per group. ** *p <* 0.01, *** p < 0.001 vs. AAV-GFP by two-way ANOVA with post hoc Tukey.

Following our visualization of α-synuclein expression in the nigrostriatal pathway, we used qRT-PCR analyses to quantify the alterations of human *α-synuclein* (*SNCA*) expression at the gene level in the nigrostriatal pathway. The expression of this gene was normalized to *β-actin* as the housekeeping gene. As there were large differences in the expression levels between animals, the data were logged for clarity of visualization. Administration of AAV-α-synuclein resulted in a significant overexpression of the human *α-synuclein* gene in the substantia nigra compared to the rats that received AAV-GFP (Figure 3a; Group, *F_(1,32)_* = 166.80, *p <* 0.05). In the striatum, the *α-synuclein* gene was similarly overexpressed (Figure 3b; Group, *F_(1,29)_* = 77.82, *p <* 0.05), with expression being localized in the nigrostriatal fibres (as visible from high magnification immunohistochemical images; data not shown).

### 2.3. AAV-α-Synuclein Administration Induced a Reduction in Astrocytic and Microglial Gene Expression in the Substantia Nigra

Continuing with our analyses of gene expression, we proceeded to assess and compare temporal changes in the expression of neuroinflammatory markers in the nigrostriatal pathway, as neuroinflammation has been recognised to be intricately linked to the pathophysiology of Parkinson’s disease [51,52] and to be impacted by the α-synuclein protein [53,54] and by cannabinoids [45,55].

**Figure 4:**
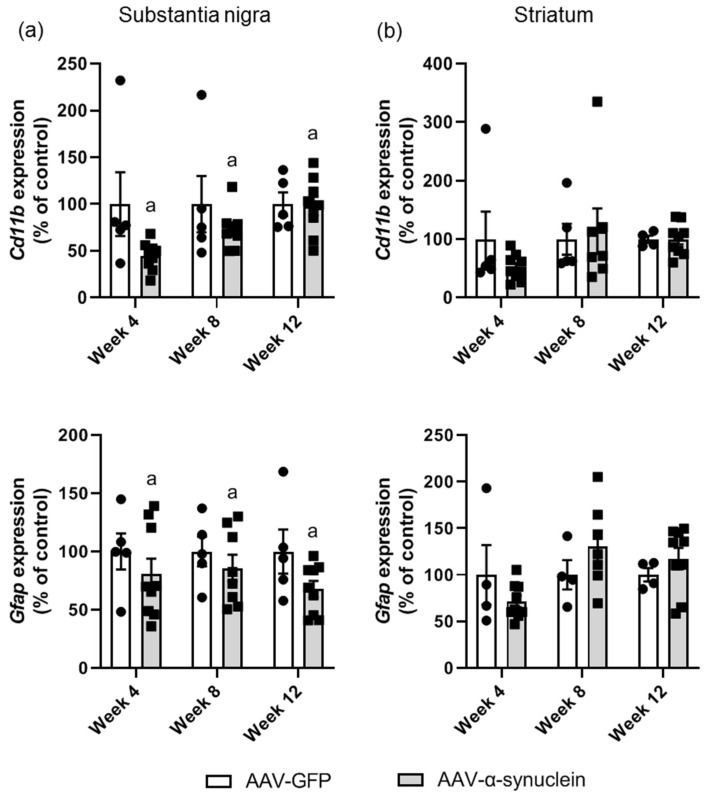
qRT-PCR analyses of astrocytic and microglial markers in the nigrostriatal pathway. In (**a**) the substantia nigra, AAV-α-synuclein administration led to an overall reduction in the expression of the microglial marker *Cd11b* and the astrocytic marker *Gfap*. (**b**) There were no differences between groups in the striatum. Data are represented as mean ± SEM with 4–9 animals per group by two-way ANOVA with post hoc Tukey. ‘a’ indicates an overall group effect.

We assessed temporal changes in the expression of the microglial marker *Cd11b* and of the astrocytic marker *Gfap* (*glial fibrillary acidic protein*), in nigral and striatal tissue by qRT-PCR. An overall reduction in mRNA levels of *Cd11b* was identified in the substantia nigra of AAV-α-synuclein animals compared to control animals (Figure 4a; Group, *F_(1,35)_* = 4.69, *p <* 0.05), although this was not found to be significant at any particular time-point in the post hoc test. In contrast, no differences in *Cd11b* were identified in the striatum (Figure 4b; Group, *F_(1,33)_* = 0.29, *p >* 0.05).

Similar results were seen with regard to the expression of the astrocytic marker *Gfap*. AAV-α-synuclein induced an overall decrease in *Gfap* expression in the substantia nigra (Figure 4a; Group, *F_(1,35)_* = 4.16, *p <* 0.05), but this did not reach significance at any particular time-point in the post hoc Tukey test. Due to the dual populations that we observed in the AAV-α-synuclein-treated rats at some time-points, we carried out correlation analyses to determine if the relative *Gfap* mRNA levels correlated with the *α-synuclein* mRNA levels. We found a significant correlation (r = 0.43, *p* < 0.005) with a seemingly biphasic response, with low levels of *α-synuclein* causing a reduction in *Gfap* expression and high levels of α-synuclein causing an increase in *Gfap* expression. No alterations were detected in the expression levels of *Gfap* in the striatum (Figure 4b; Group, *F_(1,31)_* = 0.26, *p >* 0.05).

### 2.4. AAV-α-Synuclein Did Not Alter CB_1_ or CB_2_ Receptor Expression in the Nigrostriatal Pathway

Following our analyses of the mRNA levels of the microglial and astrocytic markers, we proceeded with the primary research question of this study, which was to determine if AAV-α-synuclein induced alterations in the endocannabinoid system. The expression levels of the cannabinoid receptors, particularly the CB_2_ receptor, have been demonstrated to be altered in Parkinson’s disease patients, so we wished to determine if there were alterations induced in the gene expression of these receptors in the rat brain by this viral-mediated α-synuclein overexpression.

**Figure 5:**
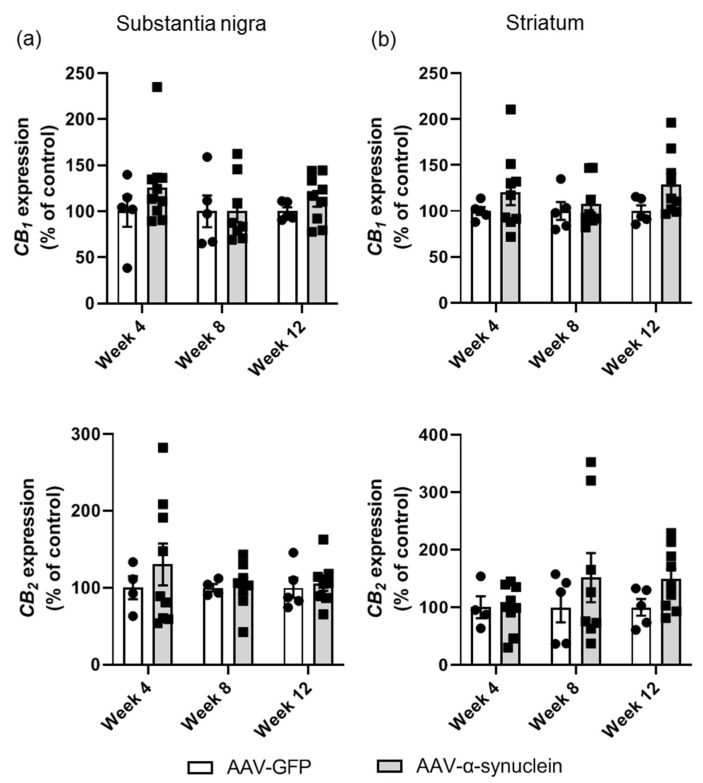
qRT-PCR analyses of cannabinoid receptor expression in the nigrostriatal pathway. There was no effect of AAV-α-synuclein expression on *CB_1_* or *CB_2_* gene expression in (**a**) the substantia nigra or (**b**) the striatum. Data are represented as mean ± SEM with 4–9 animals per group by two-way ANOVA.

In the substantia nigra, there were no differences in the levels of *CB_1_* gene expression between the two groups at any time-point (Figure 5a; Group, *F_(1,35)_* = 1.46, *p >* 0.05), and similarly, the expression of the *CB_2_* receptor gene was not altered (Figure 5a; Group, *F_(1,32)_* = 0.61, *p* > 0.05). In the striatum, there were no differences in the mRNA levels of either *CB_1_* (Figure 5b; Group, *F_(1,35)_* = 3.79, *p >* 0.05) or *CB_2_* (Figure 5b; Group, *F_(1,34)_* = 2.13, *p >* 0.05) at any time-point.

### 2.5. AAV-α-Synuclein Did Not Alter Cannabinoid Enzyme Expression in the Nigrostriatal Pathway

In addition to investigating the alterations in the expression of the endocannabinoid receptors, we also wished to assess the expression of endocannabinoid enzymes to determine if alterations in these enzymes involved in degradation were altered with α-synuclein overexpression.

**Figure 6:**
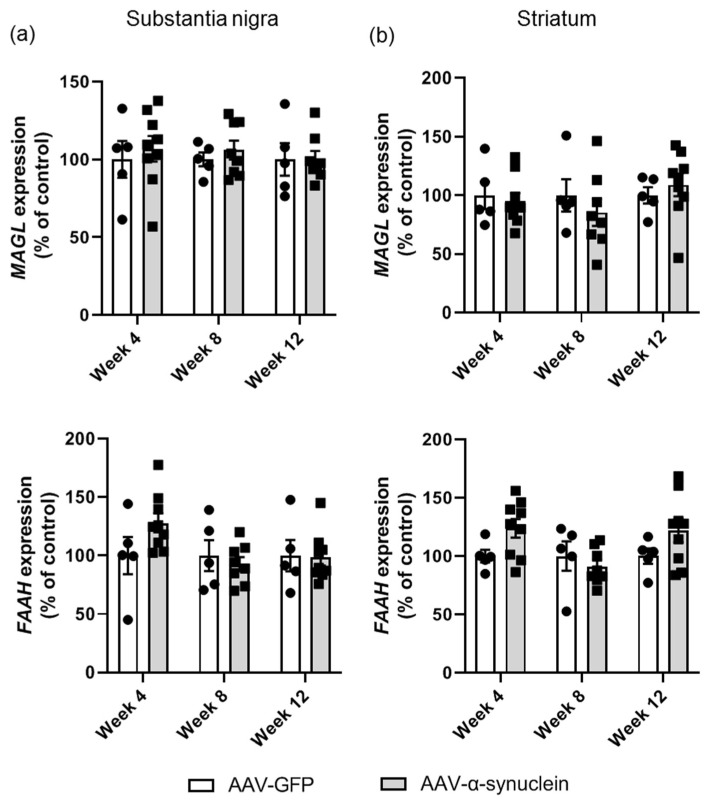
qRT-PCR analyses of the expression of endocannabinoid degrading enzymes in the nigrostriatal pathway. Administration of AAV-α-synuclein did not induce alterations in the expression of *MAGL* or *FAAH* in (**a**) the substantia nigra or (**b**) the striatum. Data are represented as mean ± SEM with 5–9 animals per group by two-way ANOVA.

This analysis revealed that administration of AAV-α-synuclein did not alter mRNA levels of *monoacylglycerol lipase (MAGL),* the 2-AG degrading enzyme, in either the substantia nigra (Figure 6a; Group, *F_(1,34)_* = 0.44, *p >* 0.05) or the striatum (Figure 6b; Group, *F_(1,35)_* = 0.17, *p >* 0.05). There was a trend for an increase in the mRNA levels of *fatty acid amide hydrolase (FAAH)*, the anandamide degrading enzyme, at some time-points in the AAV-α-synuclein group, but this was not significant in either brain region (Figure 6; substantia nigra: Group, *F_(1,35)_* = 0.60, *p >* 0.05; striatum: Group, *F_(1,35)_* = 2.69, *p >* 0.05).

### 2.6. AAV-α-Synuclein Administration Reduced the Striatal Levels of the Endocannabinoid 2-AG and the Related Lipid Mediator OEA

**Figure 7:**
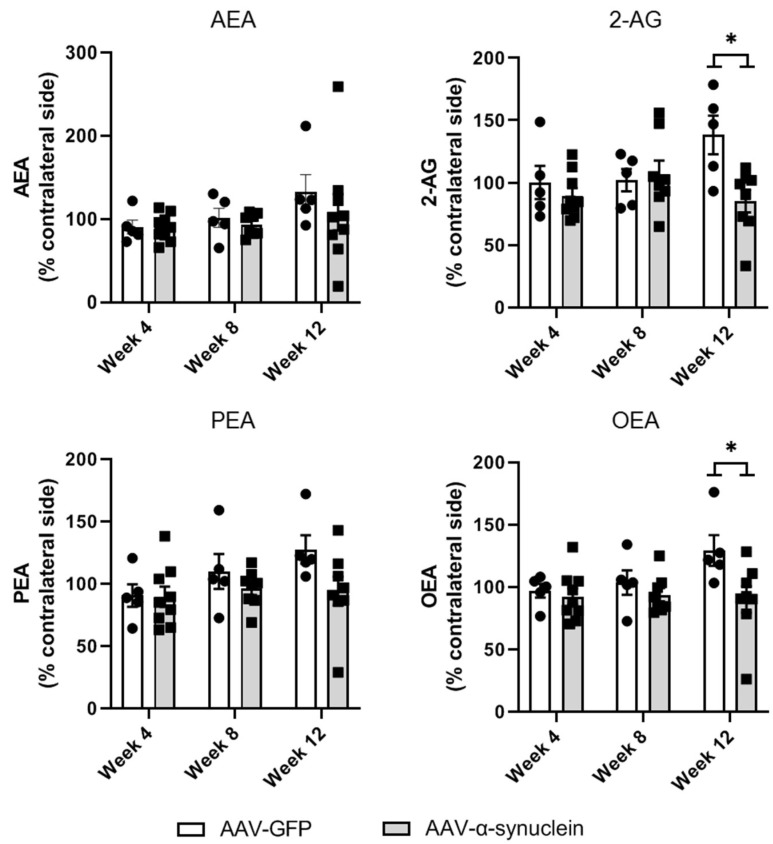
LC-MS/MS measurements of striatal endocannabinoid levels. Unilateral intranigral AAV-α-synuclein did not alter striatal AEA or PEA levels but induced a reduction in the levels of 2-AG and OEA at Week 12. Data are analysed as mean ± SEM with 5–9 animals per group. * *p* < 0.05 vs. corresponding AAV-GFP group by two-way ANOVA with post hoc Tukey.

Further to our analyses of the expression of components of the cannabinoid system at the genomic level, we sought to investigate if the intranigral administration of AAV-α-synuclein altered striatal endocannabinoid levels. To do so, we measured striatal levels of the endocannabinoids AEA and 2-AG as well as the related lipid immune mediators palmitoylethanolamide (PEA) and oleoylethanolamide (OEA) by liquid chromatography–tandem mass spectrometry (LC-MS/MS). Striatal levels of AEA or PEA (Figure 7; AEA: Group, *F_(1,35)_* = 0.83, *p* > 0.05; PEA: Group, *F_(1,34)_* = 3.69, *p* > 0.05) were not altered by AAV-α-synuclein administration. However, at Week 12, AAV-α-synuclein induced a significant decrease in the levels of the lipid mediators 2-AG (Figure 7; Group, *F_(1,34)_* = 5.17, *p* < 0.05) and OEA (Figure 7; Group, *F_(1,34)_* = 6.17, *p* < 0.05).

## 3. Discussion

Since the discovery of α-synuclein as the main component of the pathological Lewy body inclusions present in the Parkinsonian brain in 1997 [2], research into this protein has increased enormously. Yet, thus far, still very little is known about the links between this protein and the endocannabinoid signalling system. The endocannabinoid system is being investigated as a potential therapy for Parkinson’s disease from a number of avenues, including as an alleviator of motor symptoms, as an alleviator of drug-induced side effects and as a disease-modifying target with regard to providing neuroprotective or antineuroinflammatory effects [32,45]. However, it is imperative that the relationship between the α-synuclein protein and the cannabinoid system is further elucidated in order for cannabinoid-based therapy to potentially become feasible for the treatment of Parkinson’s disease in the future.

Therefore, this study sought to investigate the temporal alterations in the expression of cannabinoid system components in the rat brain in which α-synuclein is overexpressed. We assessed the expression of α-synuclein, of neuroinflammatory markers and of cannabinoid genes for a period of up to 12 weeks post-surgery. We also carried out mass spectrometry to assess striatal endocannabinoid levels and immunohistochemistry to confirm the presence of α-synuclein in the nigrostriatal pathway at the protein level.

As anticipated, we found that intranigral delivery of AAV-α-synuclein induced widespread overexpression of human α-synuclein in the nigrostriatal pathway, both at the mRNA level and the protein level. This was associated with an overall reduction in the mRNA levels of microglial and astrocytic markers in this brain region. However, despite the profound overexpression of α-synuclein and the neuroinflammatory dysregulation seen in the substantia nigra, we detected no differences in the gene expression of the *CB_1_* or *CB_2_* receptors or of the endocannabinoid-degrading enzymes *MAGL* and *FAAH*.

Further to our PCR analyses, we also carried out LC-MS/MS measurements of endocannabinoids in the striatum to determine if the surgery induced alterations in the levels of these lipid neurotransmitters at the nerve terminals. At Week 12 post-surgery, we found that α-synuclein overexpression induced a reduction in the levels of the endocannabinoid 2-AG and the related lipid immune mediator OEA compared to the control group, indicating that α-synuclein overexpression induced dysregulation of the endocannabinoid system. However, it is unclear if this is a true decrease in endocannabinoid levels, or rather a prevention of an increase, as the levels of all four endocannabinoids seem to be increasing slightly over time in the control group. Whether this is perhaps age-mediated or is induced by the overexpression of GFP is unclear. Clinically, an increase in the levels of anandamide has been reported in the cerebrospinal fluid of Parkinson’s disease patients [56,57], and similar elevations have been reported in animal models of Parkinson’s disease [58,59,60]. Increases in 2-AG have been described in the brains of several animal models of the disease [50,60,61,62], but levels were unaffected in other models [49,58,59]. Contrastingly, a decrease in 2-AG in the plasma and cerebrospinal fluid of Parkinson’s disease patients has been observed [63]. Furthermore, treatment with dopaminergic therapy has been noted to reduce the abnormally elevated levels of these cannabinoids, indicating that the increases in endocannabinoids observed may be a compensatory mechanism, with an aim to normalise dopamine depletion [57,59,61]. Clearly, the relationship between endocannabinoids and the pathophysiology of Parkinson’s disease is complex, and further research is required to elucidate this link.

The overall reduction in the expression of the astrocytic and microglial markers in the substantia nigra was surprising, but whether this reflects a decrease in the glial cell numbers in this region cannot be concluded without proper quantitative immunohistochemical analyses, which is outside the scope of this study. The link between α-synuclein expression and glial cells is very complex and is not yet fully elucidated. The CD11b protein is part of the complement receptor 3 (CR3) complex, which is involved in phagocytic activity [64]. α-synuclein has been demonstrated previously to modulate the phagocytic function of microglia [39,65], and the reduction in *Cd11b* gene expression observed here may indicate that α-synuclein may be interfering with the phagocytic functionality of microglia. Furthermore, a triplication of the *SNCA* gene was seen to compromise phagocytosis in iPSC-derived macrophages [66]. In addition, studies have demonstrated that the activation of microglia by α-synuclein may be dependent on both the degree of aggregation of α-synuclein and the protein isoform [39,43,67]. With regard to astrocytes, the reports on astrocytic activation in Parkinson’s disease patients are highly variable [68,69,70,71]. It is not clear whether astrocytes facilitate the removal of α-synuclein by phagocytosis or contribute to its propagation [72]. Undoubtedly, much remains to be learnt concerning the link between α-synuclein and the neuroinflammatory aspect of Parkinson’s disease.

In summary, the results presented in this study depict that in the rat brain in which α-synuclein was overexpressed, there were changes induced in the neuroinflammatory system, in addition to a dysregulation of the endocannabinoid system. Further research is required to elucidate the link between the cannabinoid system and the α-synuclein pathology of Parkinson’s disease in order for cannabinoid therapies to reach the clinic.

## 4. Materials and Methods

### 4.1. Animals

A total of 56 adult female Sprague Dawley rats (born in-house at the National University of Ireland Galway, Galway, Ireland) were used in this research. All procedures involving the use of animals were in compliance with the European Union Directive 2010/63/EU and the Irish Statutory Instrument S.I. No. 543 of 2012; were approved by the Animal Care and Research Ethics Committee (ACREC) of the National University of Ireland, Galway; and were completed under project and individual authorisations issued by Ireland’s Health Products Regulatory Authority (Project Authorisation: AE19125\P078). Animals were housed in pairs on a 12:12 light:dark cycle with 19–23 °C temperature and 40–70% humidity. They were provided with water and food ad libitum throughout the experiments. All post-mortem analyses were completed by a researcher blinded to the treatment of the rats.

### 4.2. Experimental Design

Rats were randomly assigned to receive dual unilateral intranigral injections of either AAV-GFP or AAV-α-synuclein. Rats were subsequently sacrificed for qRT-PCR analyses of cannabinoid system genes, as well as for α-synuclein expression and expression of microglial and astrocytic markers at 4, 8 and 12 weeks post-surgery (AAV-GFP: n = 5 per time-point; AAV-α-synuclein: n = 8–9 per time-point; power calculations were used to determine group sizes, which were based on typical variances observed in qRT-PCR data in our laboratory [49,50]; 12 weeks was chosen as the final time-point based on previous studies using this viral vector in our laboratory and others [73,74]). In addition, 2 rats (AAV-GFP) or 3 rats (AAV-α-synuclein) were sacrificed per time-point for histological visualization of α-synuclein expression in the substantia nigra and the striatum.

### 4.3. AAV Virus Production

AAV2 recombinant genomes encoding A53T human α-synuclein or GFP (green fluorescent protein) under the transcriptional control of the PGK1 (mouse phosphoglycerate kinase) promoter were pseudotyped in serotype 6 capsids as previously described [73,75]. In brief, viral particles were produced by cotransfection of HEK-293T cells with an adenovirus helper plasmid (pXX6-80), an AAV packaging plasmid carrying the rep2 and cap6 genes and a plasmid encoding the recombinant AAV2 genome containing the transgene expression cassette. Seventy-two hours after transfection, viral particles were purified and concentrated from cell lysate and supernatant by ultracentrifugation on an iodixanol density step gradient, followed by dialysis against PBSMK buffer (0.5 mM MgCl_2_ and 1.25 mM KCl in PBS). The concentration of vector stocks was estimated by real-time PCR and expressed as vector genomes per μL of concentrated stocks (vg/μL). On the day of surgery, the vectors were diluted in PBS with 0.01% Pluronic F-68 to the appropriate titre.

### 4.4. Surgery

All surgery was performed under aseptic conditions under isoflurane anaesthesia (5% in O_2_ for induction, ~2% in O_2_ for maintenance) in a stereotaxic frame with the nose bar set at −2.3 mm. All rats received a dual intranigral injection unilaterally in the substantia nigra (at the coordinates AP −4.8 and −5.8, ML +2.0 and DV −7.2) of AAV-α-synuclein or AAV-GFP at a titre of 1.33 × 10^10^ vg/µL. Infusions were carried out at a rate of 0.5 µL/min with a total volume of 3 µL per site (2 sites per rat), and a further 5 min were then allowed for diffusion.

### 4.5. Euthanasia and Tissue Processing

For immunohistochemical analyses, animals were euthanized by transcardial perfusion-fixation under terminal pentobarbital anaesthesia (Dolethal, 50 mg/kg, Vetoquinol UK Ltd, Towcester, UK). Brains were post-fixed in 4% paraformaldehyde for 24 h before being cryoprotected in a 30% sucrose solution with 0.1% sodium azide. Serial brain sections (30 µm) were cut using a freezing sledge microtome (Bright, Cambridgeshire, UK) and collected in a series of 12. For qRT-PCR and LC-MS/MS analyses, animals were sacrificed via decapitation under isoflurane anaesthesia and brains were snap-frozen on dry ice. Coronal sections (300 μm) were taken through the striatum and the substantia nigra using a cryostat (Leica CM3050 S, Germany). These sections were then micro-dissected using a cylindrical tissue puncher (Harvard Apparatus, UK; internal diameter 2 mm). Alternate punches from the striatum were used for qRT-PCR and LC-MS/MS.

### 4.6. Quantitative RT-PCR (qRT-PCR)

AAV-α-synuclein induced changes in the expression of endocannabinoid system genes were assessed by qRT-PCR as described previously [49,50]. Specifically, gene expression of the *CB_1_* and *CB_2_* receptors was analysed, as was expression of the endocannabinoid-degrading enzymes *FAAH* and *MAGL*. Expression of microglial and astrocytic markers, *Cd11b* and *Gfap*, respectively, was also assessed, as was the expression of human *α-synuclein (SNCA)*. Total RNA was extracted from punched tissue using a Macherey-Nagel extraction kit (Nucleospin RNA II; Fisher Scientific, Ireland) according to the manufacturer’s instructions. RNA quality and quantity were assessed using a DeNovix DS-11 spectrophotometer (DeNovix, Delaware, USA), and all samples were normalised to a working concentration of 13 ng/μL for the substantia nigra and 21.5 ng/μL for the striatum. 25 µL of RNA was then reverse transcribed to cDNA using an Invitrogen Superscript III reverse transcriptase custom kit (Biosciences, Dublin, Ireland). TaqMan gene expression assays (Biosciences, Dublin, Ireland) containing forward and reverse primers and a FAM-labelled MGB TaqMan probe was used to quantify the mRNA of the genes of interest on a StepOnePlus Real-Time PCR System (Applied Biosystems, Waltham, MA, USA). The genes assessed and corresponding assay IDs are as follows: *SNCA* (Hs00240906_m1), *CB_1_* (Rn00562880_m1), *CB_2_* (Rn03993699_s1), *FAAH* (Rn00577086_m1), *MAGL* (Rn00593297_m1), *Cd11b* (Rn00709342_m1), *Gfap* (Rn00566603_m1), while VIC-labelled *β-actin* (4352340E, ThermoFisher, Dublin, Ireland) was used as a housekeeping gene and endogenous control. A no template control (NTC) reaction was included in all assays. Samples were run in duplicate under the following cycling conditions: 50 °C for 2 min, 95 °C for 10 min and 40 cycles of 95 °C for 15 s / 60 °C for 1 min. Relative gene expression to endogenous control was calculated using the formula 2^−ΔCt^, where ΔCt represents the magnitude of the difference between cycle threshold (Ct) values of the target and endogenous control, and the result expressed as a percentage of the mean value of the control group.

### 4.7. Liquid Chromatography–Tandem Mass Spectrometry

Quantification of the concentration of endocannabinoids and related *N*-acylethanolamines was carried out in the NUIG Biological Mass Spectrometry Core Facility (National University of Ireland Galway, Galway, Ireland) according to a standardised protocol (unpublished). Samples and standards were prepared essentially as previously described [76,77]. Specifically, levels of AEA, 2-AG, PEA and OEA were analysed. In brief, 200 μL of 100% acetonitrile containing deuterated internal standards for endogenous cannabinoid ligands (2.5 ng d8-AEA, 50 ng d8-2-AG, 2.5 ng d4-PEA, 2.5ng d4-OEA; Cayman Chemicals, Biosciences, UK) and 75 μL of 100% acetonitrile were added to the samples. The tissue was homogenized for ~3 sec using a sonicator (Branson Sonifier 150, Branson, UK) and then centrifuged at 14,000 × g for 15 min at 4 °C (Hettich^®^ Centrifuge Mikro 220R, Hettich, Germany). A standard curve 1:4 dilution was prepared; the highest standard (Standard 10) was made up by adding 25 µL of 100% acetonitrile containing a known fixed amount of nondeuterated internal standard (12.5 ng AEA, PEA, OEA and 125 ng 2-AG) to 75 µL of 100% pure acetonitrile solution. Finally, 200 µL of 100% acetonitrile containing a known fixed amount of deuterated internal standard was added to each standard. 40 μL of each sample and standard curve point were added to HPLC vials.

Mobile phases consisted of solution A (HPLC grade water with 0.1% (v/v) formic acid) and solution B (100% acetonitrile with 0.1% (*v*/*v*) formic acid). These solutions were maintained at a flow rate of 0.3 mL/min, injected onto a Zorbax^®^ SB C18 column (Agilent Technologies, Santa Clara, CA, USA) with length, internal diameter and particle size dimensions of 50 mm, 2.1 mm and 1.8 μm, respectively. Reversed-phase gradient elution was used, comprising of 45% Solution B for the first minute, then linearly increased to 100% until 5 min into the run, which was then maintained until the assay run finished at 15 min. A further 5 min was used to re-equilibrate the column at 45% Solution B before the next injection. Under these conditions, AEA, 2-AG, PEA and OEA were eluted at the following retention times: 6.7 min, 7.0 min, 7.1 min and 7.3 min, respectively. Analyte detection was carried out in electrospray-positive ionization mode on an Agilent 1260 infinity 2 HPLC system coupled to a SCIEX QTRAP 4500 mass spectrometer operated in triple quadrupole mode (SCIEX Ltd., Macclesfield, UK). Instrument conditions were optimized for each analyte by infusing standards separately. Ratiometric quantification was performed using Skyline Quantitative Analysis Software version 21.1 (MacCoss Lab Software, University of Washington, WA, USA). The amount of analyte in unknown samples was calculated from the analyte/internal standard peak area response ratio using a 10-point calibration curve constructed from a range of concentrations of the nondeuterated form of each analyte and a fixed amount of deuterated internal standard.

### 4.8. Immunohistochemistry

Free-floating immunohistochemistry (IHC) was performed using the streptavidin-biotin-peroxidase method as previously described [49,78]. In brief, sections were quenched in a solution containing 3% hydrogen peroxide and 10% methanol in distilled water to eliminate endogenous peroxidase activity. Nonspecific antibody binding was blocked by incubation in a solution containing 3% normal goat serum in Tris-buffered saline (TBS) with 0.2% Triton X-100 at room temperature for 1 h. The primary antibody (mouse antihuman-α-synuclein, 1:10,000, 36-008, Merck Millipore, Cork, Ireland) was diluted in 1% serum in TBS with 0.2% Triton X-100 and allowed to incubate with the sections overnight. Sections were then incubated with the biotinylated secondary antibody (goat antirabbit, 1:200, 111-065-144, Jackson ImmunoResearch, Cambridgeshire, UK) with 1% serum for 3 h. A streptavidin-biotin-horseradish peroxidase solution (Vector PK-4000) was subsequently added to sections and allowed to incubate for 2 h. Development of the staining was performed using a 0.5% diaminobenzidine tetrahydrochloride (DAB) (Sigma D5637) solution in TBS containing 0.3 µL/mL of hydrogen peroxide. Sections were mounted onto gelatine-coated slides, dehydrated in an ascending series of alcohols, cleared in xylene and coverslipped using DPX mountant.

### 4.9. Statistical Analysis

Statistical analyses were carried out using GraphPad Prism software (GraphPad Software, San Diego, CA, USA). Prior to analyses, data were tested for normality using a Shapiro–Wilk test and homogeneity of variance using a Brown–Forsythe test to determine if the data were parametric or nonparametric. All parametric data were expressed as mean ± standard error of the mean (SEM). Two-way ANOVA was used to compare the means of two or more groups on two factors simultaneously, with post hoc Tukey analysis. Throughout the results, the main outcome from the ANOVA is given in the text while the outcome of any post hoc analysis is shown in the relevant figure and explained in the corresponding legend (except in Figure 4, where “a” in the figure indicates an overall group effect from the ANOVA). Linear regression analyses were used to determine if there was any correlation between human *α-synuclein* expression and *Gfap* expression. In all cases, results were deemed significant if *p* < 0.05.

## Data Availability

Research data used in this article are available from the corresponding author on request.

## Sample Availability

Samples are not available from the authors.
